# Dataset of agromorphological traits in early population of turmeric (*Curcuma longa* L.) local accessions from Indonesia

**DOI:** 10.1016/j.dib.2020.106552

**Published:** 2020-11-21

**Authors:** Putri Ardhya Anindita, Tresna Kusuma Putri, Debby Ustari, Haris Maulana, Meddy Rachmadi, Vergel Concibido, Tarkus Suganda, Agung Karuniawan

**Affiliations:** a Faculty of Agriculture, Universitas Padjadjaran, Bandung, Indonesia; b Bioresources Management, Graduate School, Universitas Padjadjaran, Bandung, Indonesia; c PhD. Students, Faculty of Agriculture, Universitas Padjadjaran, Bandung, Indonesia; d Sensient Colors, LLC, 2515 North Jefferson Avenue, St. Louis, MO, United States

**Keywords:** Agronomic traits, Genetic resources, Indonesia, Morphological traits, Phenotypic diversity, Turmeric

## Abstract

Turmeric (*Curcuma longa* L.) is widely used as traditional medicine, kitchen spice, and natural dyes in Indonesia. The demand and popularity of this plant is increasing; however, the national supply is still low. Turmeric breeding and crop improvement in Indonesia are needed to boost the national turmeric production. Exploration of turmeric from various areas in Indonesia is a prior step of turmeric breeding. Phenotypic diversity and relationship analysis of an early population can be used as a basis for consideration in plant development and breeding programs. The purpose of this study was to estimate phenotypic diversity and relationship of local turmeric accessions from Indonesia. Raw data analysis was conducted after standardization using Principal Component Analysis (PCA) and Hierarchical Clustering Analysis (HCA) to determine phenotypic diversity and relationship among the newly collected genetic resources. The data in this article showed broad phenotypic diversity with plant height, number of shoots, number of leaves on main shoot, petiole length, lamina length, lamina width, number of mother rhizome, total rhizome weight, weight per shoot, pseudostem habit, leaf margin, and rhizome habit as distinguishing traits among the collection. PCA also resulted cumulative variation percentage of 70.73%. In addition, HCA resulted two main groups with the Euclidean range of 0.31—2.97.

**Specifications Table**

**Table:** 

Subject	Data Article (Agricultural and Biological Science)
Specific subject area	Agricultural and Biological Science (general), Agronomy and Crop Science
Type of data	Table Figure
How data were acquired	Raw data were acquired as the result of observation, measurements, and sampling in the field and after harvesting. Rhizome color were determined using Royal Horticultural Society Colour Chart (RHSCC). Instrument: Microsoft Excel 2010, NTSyspc version 2.11x
Data format	Analysed
Parameters for data collection	The conditions considered for data collection were specified time of observation and environmental conditions of the experiment.
Description of data collection	Data was collected by measuring 27 agromorphological traits at the specified times in planting location. Five plant samples were taken from one plot (accession). Data collection was carried out at 150 days after planting for upper plant observation and after harvesting which occurred 11 months after planting for rhizome observation.
Data source location	Ciparanje Experimental Field Institution: Universitas Padjadjaran City/Town/Region: Sumedang Regency, West Java Country: Indonesia Latitude and longitude for collected samples/data: 6°55′0.72804″ S latitude and 107°46′18.46056″ E longitude The mean temperature was 23.2 °C and the mean humidity was 90.3%.
Data accessibility	With the article

## Value of the Data

• This dataset provides information about the estimated phenotypic diversity and relationship of local turmeric germplasms from Indonesia.
• The dataset in this article provides information about the defining characteristics among Indonesian local turmeric accessions studied. The information can be used further for research, crop production, and/or industrial purposes.
• The dataset provided in this article can be used in genetic studies and breeding programs of turmeric, especially the assembly of new varieties targeted to industrial purposes specified in natural dyes, spices, herbal based health products, and others.

## 1 Data Description

**Fig. 1:**
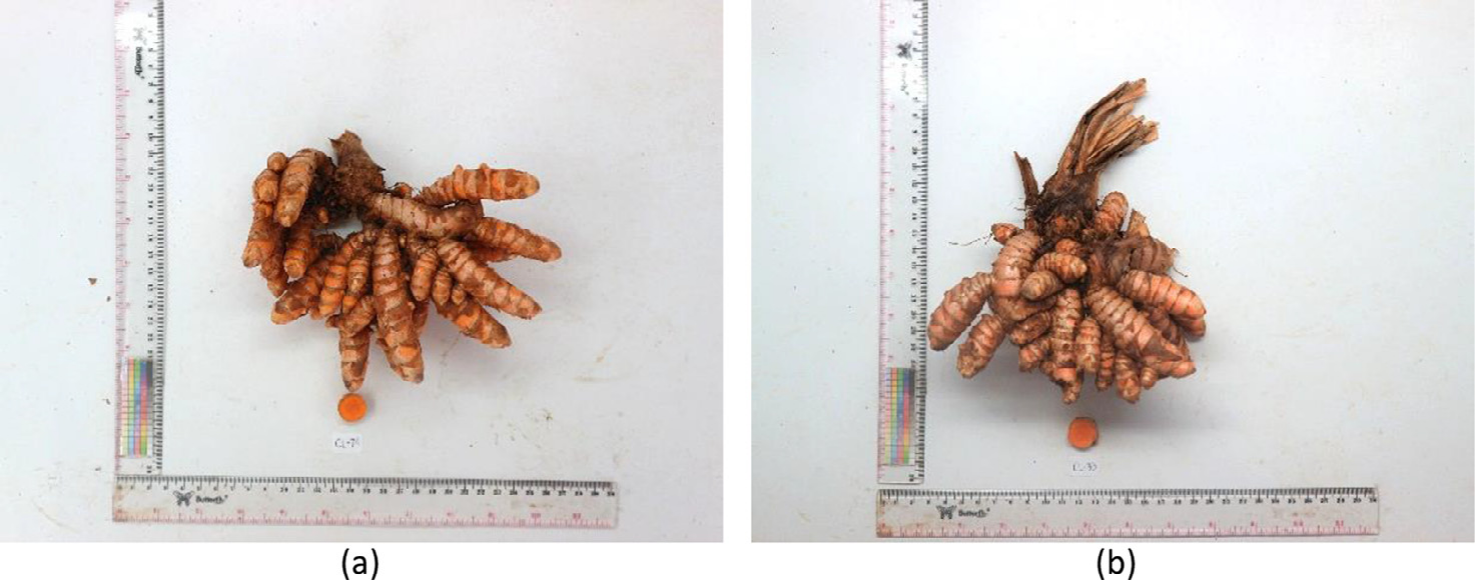
Rhizome appearance of turmeric: (a) CL-JMB-01 from Jambi; (b) CL-SUT-03 from South Sumatra.

**Fig. 2:**
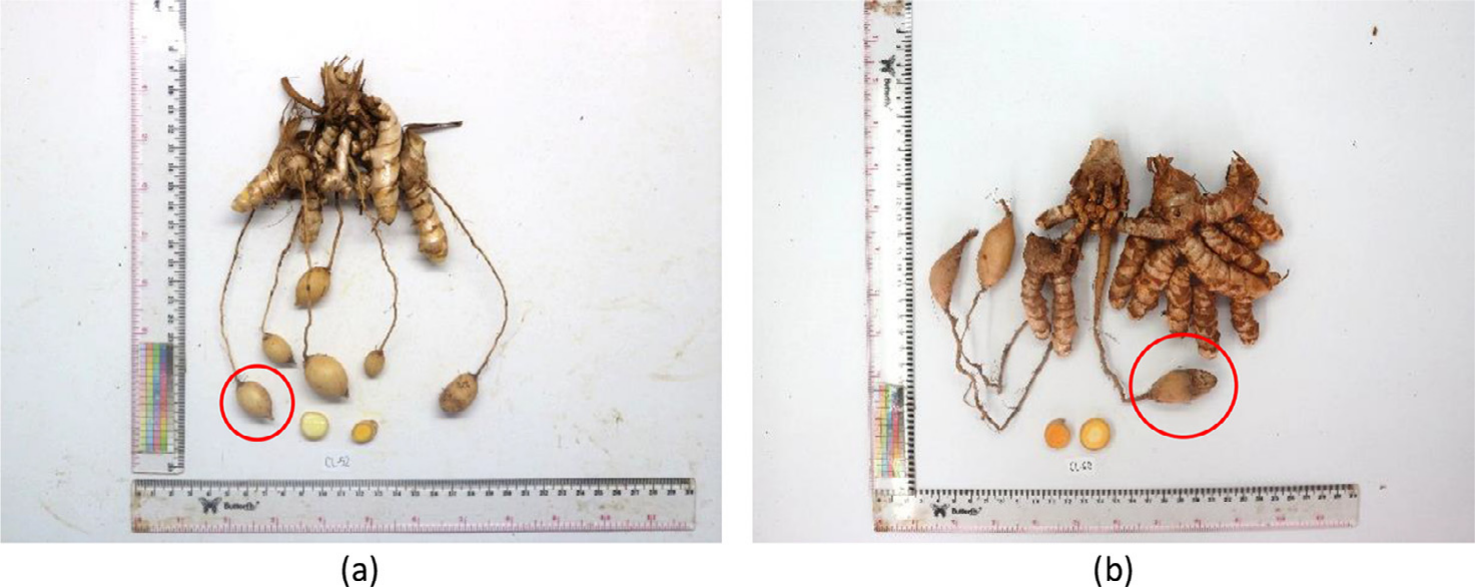
Rhizome appearance of turmeric: (a) CL-PPB-04 from West Papua; (b) CL-PPB-12 from West Papua. T-roots were showed in red circles.

This data article contains data from 91 turmeric accessions (Table 1) with 27 agromorphological traits observed. The traits were composed of 15 quantitative and 12 qualitative traits which were described in Table 2. Observation result showed some unique characteristics that is diverse in different provinces and islands. For example, turmeric from Jambi (Fig. 1a) and South Sumatra (Fig. 1b) have deep orange, straight, and bigger rhizome, while in Fig. 2 turmeric accessions from West Papua have bright yellow, curved, and smaller rhizome, also it had the appearance of tuber-like roots known as T-roots [1]. Similar studies were previously conducted with the result of high diversity of turmeric germplasms in specific areas based on agromorphological traits. Another study also reported that high diversity based on agromorphological traits was showed in 20 turmeric germplasms from three differents ecological zones in Pakistan [2] and 83 turmeric germplasms in the North-eastern India regions [3].

**Table 1:** List of accessions used in this study.

Code	Origin (Province)	Number of Accessions
CL-JBR	West Java	15
CL-JTG	Central Java	3
CL-JTM	East Java	6
CL-BTN	Banten	2
CL-NAD	Aceh	2
CL-SUT	North Sumatra	4
CL-SSL	South Sumatra	4
CL-SMB	West Sumatra	1
CL-JMB	Jambi	3
CL-BKL	Bengkulu	2
CL-LMP	Lampung	3
CL-BKL	Bangka Belitung Islands	5
CL-KLB	West Kalimantan	1
CL-KLT	East Kalimantan	1
CL-KTG	Central Kalimantan	1
CL-SLS	South Sulawesi	4
CL-SLT	Southeast Sulawesi	2
CL-SLU	North Sulawesi	1
CL-STG	Central Sulawesi	2
CL-GTL	Gorontalo	1
CL-BAL	Bali	2
CL-MLK	Maluku	4
CL-MUT	North Maluku	1
CL-NTB	West Nusa Tenggara	2
CL-PAP	Papua	2
CL-PPB	West Papua	11
Check accession		
CHECK 1	West Java	1
CHECK 2	West Java	1
CHECK 3	West Java	1
CHECK 4	West Java	1
CHECK 5	Special Region of Yogyakarta	1
CHECK 6	Lampung	1
	Total	91

**Table 2:** List of traits observed in this study.

Trait	Abbreviation	Type
Pseudostem habit	PsH	Qualitative
Plant height	PlH	Quantitative
Number of shoots	NS	Quantitative
Number of leaves per main shoot	NLMS	Quantitative
Leaf disposition	Ldi	Qualitative
Petiole length	PL	Quantitative
Lamina length	LL	Quantitative
Lamina width	LW	Quantitative
Leaf dorsal color	Ldo	Qualitative
Venation pattern	VP	Qualitative
Leaf Margin	LM	Qualitative
Coma bract color	CBC	Qualitative
Bract tip color	BTC	Qualitative
Rhizome habit	RH	Qualitative
Rhizome shape	RS	Qualitative
Primary rhizome length	PRL	Quantitative
Primary rhizome width	PRWi	Quantitative
Primary rhizome thickness	PRT	Quantitative
Rhizome width	RW	Quantitative
Number of mother rhizomes	NMR	Quantitative
Rhizome color	RC	Qualitative
Internode pattern	IP	Qualitative
Appearance of tertiary rhizome	TR	Qualitative
Total rhizome wright	TRW	Quantitative
Weight per shoot	WPS	Quantitative
Primary rhizome weight	PRWe	Quantitative
Dry recovery	…DRC	Quantitative

**Fig. 3:**
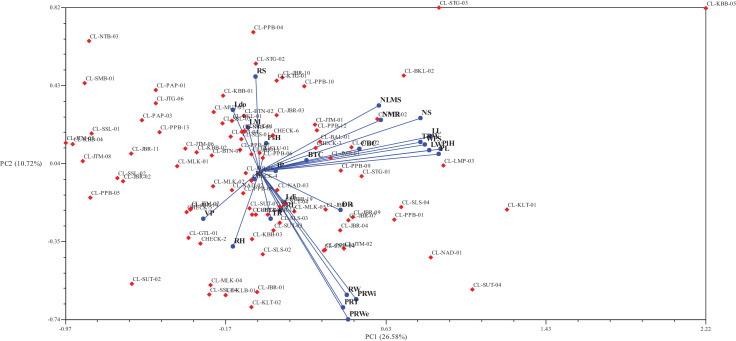
Biplot of local turmeric accessions from Indonesia based on first and second PC.

PCA resulted in some principal components (PCs) which represent group of large data and determine traits that have a contribution to the variation [4]. The amount of PC and distinguishing traits were based on eigenvalue of more than one [8] and loading factors of more than 0.5 or less than −0.5 [9]. Table 3 showed that the high variation among the analysed data based on cumulative variation percentage of 70.73%. A population was considered has high diversity if the cumulative variance percentage was more than 50% [4]. The first three PCs in the Table 3 showed that the plant height, number of shoots, number of leaves on main shoot, petiole length, lamina length, lamina width, number of mother rhizome, total rhizome weight, weight per shoot, pseudostem habit, leaf margin, and rhizome habit, as differentiator among accessions as the traits had the most variation among the observed accessions. Fig. 3 showed that the biplot of the result from PCA in two-dimensional way based on the first and second PCs. In Fig. 3, data points of turmeric accessions from each area had different appearance. Most accessions from the same origin scattered in all quadrants of the biplot, such as West Java and West Papua. Some accessions from the same origin also had a close data points that showed a possibility of both accessions being planted from the same source.

**Table 3:** Result of PCA on turmeric local accessions from Indonesia.

Trait	PC1	PC2	PC3	PC4	PC5	PC6	PC7	PC8
PsH	0.035	0.140	**0.623**	0.470	0.249	0.046	0.168	0.001
PlH	**0.903**	0.112	0.024	0.212	−0.002	0.049	−0.045	0.148
NS	**0.803**	0.263	0.016	−0.217	−0.021	0.220	−0.144	0.016
NLMS	**0.597**	0.325	0.029	0.312	−0.363	0.014	0.175	−0.115
Ldi	0.125	−0.156	0.392	0.225	0.305	−0.144	−0.494	0.186
PL	**0.895**	0.086	−0.022	0.035	−0.118	0.137	0.112	0.059
LL	**0.855**	0.172	−0.062	0.215	−0.080	−0.021	0.066	0.090
LW	**0.846**	0.103	−0.123	0.249	−0.068	−0.078	−0.006	0.155
Ldo	−0.129	0.304	−0.242	0.464	−0.180	−0.249	−0.216	−0.329
VP	−0.277	−0.239	−0.026	−0.191	−0.177	0.468	0.368	0.367
LM	−0.055	0.217	**0.664**	0.000	−0.119	−0.066	−0.262	−0.044
CBC	0.500	0.112	−0.192	−0.484	0.304	−0.136	0.097	−0.208
BTC	0.236	0.054	−0.353	0.106	**0.550**	−0.192	0.382	−0.299
RH	−0.129	−0.375	**−0.535**	0.366	0.021	0.226	−0.093	0.126
RS	−0.017	0.471	0.044	0.205	−0.226	0.016	0.383	−0.097
PRL	0.105	−0.199	−0.129	0.015	0.300	**0.517**	−0.295	−0.285
PRWi	0.485	**−0.640**	−0.112	−0.004	−0.170	−0.273	0.069	−0.117
PRT	0.418	**−0.681**	−0.046	0.013	−0.204	−0.377	0.070	−0.113
RW	0.438	**−0.619**	0.279	0.121	−0.036	−0.016	0.027	−0.013
NMR	**0.606**	0.254	−0.144	−0.023	0.110	−0.006	0.010	0.143
RC	−0.024	−0.042	−0.452	0.066	0.422	0.151	−0.477	−0.195
IP	0.083	0.001	−0.415	0.172	0.163	−0.057	−0.056	**0.596**
TR	0.057	−0.237	0.229	0.099	−0.231	0.490	0.176	−0.175
TRW	**0.803**	0.145	0.034	−0.395	−0.053	0.087	−0.116	−0.065
WPS	**0.825**	0.132	0.114	−0.339	0.033	0.035	−0.167	−0.014
PRWe	0.442	**−0.740**	0.241	−0.042	−0.037	−0.058	0.088	0.023
…DRC	0.406	−0.196	−0.006	0.383	0.405	0.338	−0.006	−0.202
Eigenvalue	7.176	2.894	2.145	1.722	1.438	1.369	1.312	1.043
Percentage (%)	26.577	10.718	7.944	6.376	5.325	5.069	4.860	3.861
Cumulative (%)	26.577	37.295	45.239	51.615	56.939	62.009	66.869	70.730

PC = Principle Component; PsH = pseudostem habit, PlH = plant height, NS = number of shoots, NLMS = number of leaves per main shoot, Ldi = leaf disposition, PL = petiole length, LL = lamina length, LW = lamina width, Ldo = leaf dorsal color, VP = venation pattern, CBC = coma bract color, BTC = bract tip color, RH = rhizome habit, RS = rhizome shape, PRL = primary rhizome length, PRWi = primary rhizome width, PRT = primary rhizome thickness, RW = rhizome width, NMR = number of mother rhizomes, RC = rhizome color, 1P = internode pattern, TR = appearance of tertiary rhizome, TRW = total rhizome wright, WPS = weight per shoot, PRWe = primary rhizome weight, … DRC = dry recovery.

**Fig. 4:**
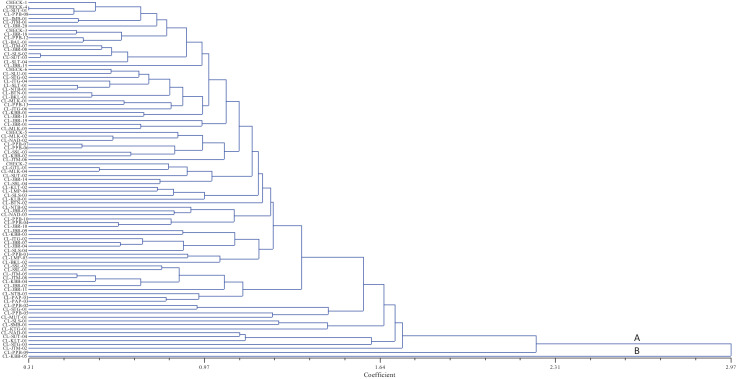
Dendrogram of local turmeric accessions from Indonesia.

Fig. 4 showed the dendrogram as the result of Hierarchical Clustering Analysis (HCA) based on agromorphological traits. Cluster analysis divides a heterogenous population into more homogenous groups, or in other words, grouping a random data items based on the similarity or dissimilarity between each data [10] and represented in the dendrogram used Euclidean dissimilarity coefficient or Euclidean distance that showed relatedness between two or more accessions based on variables used [11]. Turmeric accessions in this study were divided into two groups with the first group divided into two subgroups i.e. class A and class B. Euclidean distance was ranged from 0.31 to 2.97 which considered as the accessions has the distant relationship. According to Mc Garigal et al. (2000) [12], euclidean distance of more than 0.75 showed a distant phenotypic relationship among accessions tested. The most similar accessions were CHECK 4 from West Java and CL-SUT-01 from North Sumatra. CL-KBB-05 from Bangka Belitung Islands was the only accession in class B related to its superiority in several agronomic traits i.e. plant height, number of mother rhizomes, and yield. The superiority was also corroborated with the accession's data point position in the biplot which was quite far from the center point and other data points.

## 2 Experimental Design, Materials and Methods

### 2.1 Plant materials

The study in this data article used 85 turmeric accessions and six check accessions from the collection of Laboratory of Plant Breeding and Seed Technology, Universitas Padjadjaran. The accessions were newly obtained from various areas in Indonesia with a total of 26 provinces and had not been studied before.

### 2.1 Experiment location

All the accessions were planted at Ciparanje Experimental Field, Faculty of Agriculture, Universitas Padjadjaran from August 2018 to July 2019. The site was located at the altitude of 753 m above sea level and on 6°55′0.72804″ S latitude and 107°46′18.46056″ E longitude. The soil type was described as inceptisols with clay texture. The mean temperature during this study was 23.2 °C and the mean humidity was 90.3%. The cultivation started at the end of dry season and rainy season occurred after two months of cultivation. The total rainfall during this study was 1800 mm per year.

### 2.2 Experimental design and planting

This study used augmented design with three blocks and six check accessions as control to adjust the availability of each accession. Each accession was planted in one row with ten plants on each row. The plant spacing was 0.5 × 1 m. Before planted, the turmeric rhizomes were sown used a 2:1 ratio of husk charcoal and cocopeat media until the rhizome sprouted and developed leaves. The sprouted rhizomes were transplanted in the field at a mounds as high as 20 cm. The soil had been treated with chicken dunk with a dosage 45 t/ha seven days before planting. Bion-Up organic fertilizer solution was applied at a rate of 10 mL per 1 L of water one month after transplanted. The Harvested time was conducted 11 months after planted or when the whole part of plant (leafs dan stems) was dry out [5].

### 2.3 Data collection

The data in this study was collected on 150 days after planted and after harvested time. The data was collected by measured agromorphological traits based on Guideline for the Conduct of Test for Distinctiveness, Uniformity, and Stability from Protection of Plant Varieties and Farmers' Rights Authority, India [6]. Five plant samples were taken from each plot (accession) for measured and observed. The tools used were digital scale, measure tape, calliper, and color chart using Royal Horticultural Society Colour Charts.

### 2.4 Data analysis

Estimation of phenotypic diversity was conducted used a Principal Component Analysis (PCA) follows Mooi et al. (2018) [4] and Hierarchical Clustering Analysis was conducted to estimated the phenotypic relationship follows Morissette and Chartier (1967) [7]. Both analysis were performed using NTSyspc version 2.11x.

## Declaration of Competing Interest

The authors declare that they have no known competing financial interests or personal relationships which have, or could be perceived to have, influenced the work reported in this article.

## References

[1] Lobo R., Prabhu K.S., Shirwaikar A., Shirwaikar A. (2008). Curcuma zedoaria Rosc. (white turmeric): a review of its chemical, pharmacological and ethnomedicinal properties. J. Pharm. Pharmacol..

[2] Jan H.U., Rabbani M.A., Shinwari Z.K. (2012). Estimation of genetic variability in turmeric (Curcuma longa l.) germplasm using agro-morphological traits. Pakistan J. Bot..

[3] Roy S., Misra A.K., Verma S.K., Singh S.K. (2011). Agro-morphological diversity in turmeric (Curcuma longa) accessions collected from north-eastern India. Indian J. Agric. Sci..

[4] Mooi E., Sarstedt M., Mooi-Reci I. (2018). Market Research.

[5] Nair K.P. (2019). Turmeric (Curcuma Longa L.) and Ginger (Zingiber Officinale Rosc.) - World's Invaluable Medicinal Spices.

[6] Indian Institute of Spices Research, “Guidelines for conduct of test for distinctiveness, uniformity and stability on turmeric (Curcuma longa L.),” 2007. [Online]. Available: http://www.plantauthority.gov.in/pdf/turmeric.pdf.

[7] Morissette L., Chartier S. (2013). The k-means clustering technique: general considerations and implementation in Mathematica. Tutor. Quant. Methods Psychol..

[8] Jeffers J.N.R. (1967). Two case studies in the application of principal component analysis. Appl. Stat..

[9] Wiktorowicz J. (2016). Exploratory factor analysis in the measurement of the competencies of older people. Ekonometria.

[10] Murtagh F., Contreras P. (2011). Methods of hierarchical clustering. Int. Encycl. Stat. Sci..

[11] Liberti L., Lavor C., Maculan N., Mucherino A. (2014). Euclidean distance geometry and applications. SIAM Rev.

[12] McGarigal K., Cushman S., Stafford S. (2000). Multivariate Statistics for Wildlife and Ecology Research.

